# Trauma in 2012

**DOI:** 10.1186/1757-7241-21-S1-A8

**Published:** 2013-05-28

**Authors:** L Salm, CE Hommers

**Affiliations:** 1 Bristol, UK; 2 Bath, UK

## 

The Trauma Conference comprised of an international program encompassing civilian and military expertise in topical areas of trauma management from both a clinical and a systems-based perspective. Speakers challenged outdated systems or beliefs and presented alternatives in this evolving area of medicine.

The conference opened with three speakers on the subject of severe pelvic trauma. Mr Mark Rickman, an Orthopaedic Surgeon from London covered the *Perspective of the Orthopaedic Surgeon*. In a review of the epidemiology and pathophysiology he reminded us of the high-energy nature of these injuries and the need to look for, and treat, associated injuries. Complex pelvic trauma, where a fracture is combined with open skin or visceral injury carries a mortality of 19 - 27%. He re-iterated the importance of senior multi-disciplinary input and decision-making. The use of external fixators is an outdated practice - a role adequately fulfilled by the application of a simple pelvic binder. On questioning he stated that binders could remain in place for up to 24 – 36 hours until haemodynamic stability was maintained and for definitive fixation to be carried out between 3 and 7 days. He highlighted the 10% association with urogenital injury, which itself can have significant influence on mortality (14% vs. 8%). He suggested it was safe for a single cautious attempt at catheterisation in these patients but that they should all undergo a cystourethrogram to exclude injury.

Mr Jan Jansen, consultant in General Surgery and Intensive Care Medicine from Aberdeen Royal Infirmary, gave us his perspective on *The Role of the General Surgeon* in these patients. He opened by attempting to dispel the myth that one should ‘never operate on a patient with a pelvic fracture and a pelvic haematoma’. He went on to explain that the majority of pelvic bleeds are venous in origin with a low incidence of arterial bleeding. Angiography and embolisation are predominately effective for arterial bleeding, and in context of the practical and logistical constraints of accessing these services acutely, he highlighted the limited role radiology may have to play. Echoing the previous speaker Mr Jansen recommended keeping stabilisation of the pelvic fracture simple with a pelvic binder and does not believe that the presence of a binder would prevent the performance of a damage control laparotomy. He discussed the role of different types of pelvic packing and the growing clinical experience and evidence for its role in control of venous bleeding, including the Eastern Groups Guidelines for the management of haemorrhage in pelvic fracture [1]. Whilst there may be a role for complimentary approaches of pelvic packing and angiography (where immediately available) he concluded that in his practice the unstable patient with a pelvic fracture requires urgent pelvic packing with or without bilateral internal artery ligation for inflow control.

Next our military colleagues presented a series of lectures on the lessons learned from conflict medicine. The sad reality is that there have been a huge number of casualties in recent conflicts, most notably Afghanistan. As in the past, this has led to significant technical and conceptual advances in medicine, especially in our understanding and approach to trauma and is saving soldiers lives as well as guiding the provision of trauma care in civilian populations. Wing Commander Jon Kendrew, a consultant Orthopaedic Surgeon in Birmingham, summarised the underlying principal of the military approach to major trauma as restoring physiology not anatomy by performing minimum rapid essential surgery. He emphasised the importance of understanding the physiology of trauma and the key role that training in damage control resuscitation and surgery has on outcome. He underpinned this debate by showing that only 1 in 10 final year trainees and new consultants are truly confident with delivering safe and effective haemostatic control.

The first day was closed by Lt Col Rhys Thomas an army consultant anaesthetist with 16 Air Assault Medical Regiment, attributed the current three-fold higher rate of unexpected survivors in Afghanistan to this military team approach to damage control surgery and resuscitation, as well as improved understanding of the pathophysiology of trauma. He discussed practical aspects of effective advanced damage control shock resuscitation including: availability of shock packs, avoidance of crystalloid, high ratio resuscitation, tranexamic acid, point of care testing and goal directed therapy, the utilisation of fresh blood components, prevention of peripheral hypoperfusion by the avoidance of vasopressors, the use of algorithms and senior clinical input with early aggressive decision making.

